# Characterization of Eco-Friendly Fabricated and Induction-Sintered Hydroxyapatite-Based Hybrid Composites

**DOI:** 10.3390/ma18235359

**Published:** 2025-11-28

**Authors:** Esra Nur Usta, Nermin Demirkol, Bilgehan Cem Turan, Mevlüt Gürbüz, Gültekin Göller, Katia Barbaro, Daniele Sagrafoli, Marco Fosca, Julietta V. Rau

**Affiliations:** 1 Department of Biomedical Engineering, Faculty of Technology, Kocaeli University, 41001 Kocaeli, Türkiye; sarikayaes44@gmail.com; 2 Department of Ceramics, Faculty of Fine Arts, Kocaeli University, 41300 Kocaeli, Türkiye; 3 Department of Mechanical Engineering, Faculty of Engineering, Ondokuz Mayıs University, 55139 Samsun, Türkiye; bilgehancemturan@gmail.com (B.C.T.); mgurbuz@omu.edu.tr (M.G.); 4 Department of Metallurgical and Materials Engineering, Faculty of Chemistry and Metallurgy, Istanbul Technical University, 34467 Istanbul, Türkiye; goller@itu.edu.tr; 5 Istituto Zooprofilattico Sperimentale Lazio e Toscana “M. Aleandri”, Via Appia Nuova 14111, 00178 Rome, Italy; katia.barbaro@izslt.it (K.B.); daniele.sagrafoli@izslt.it (D.S.); 6 Istituto di Struttura della Materia, Consiglio Nazionale delle Ricerche, ISM-CNR, Via del Fosso del Cavaliere 100, 00133 Rome, Italy; marco.fosca@ism.cnr.it (M.F.); giulietta.rau@ism.cnr.it (J.V.R.); 7 Department of Analytical, Physical and Colloid Chemistry, Institute of Pharmacy, Federal State Autonomous Educational Institution of Higher Education I.M. Sechenov First Moscow State Medical University of Ministry of Health of the Russian Federation (Sechenovskiy University), Trubetskaya 8, Build. 2, 119048 Moscow, Russia

**Keywords:** induction sintering, sheep hydroxyapatite, green process, magnesium oxide, graphene, bioactivity, cell culture

## Abstract

In this study, eco-friendly sheep hydroxyapatite (KHO) powder was produced from sheep femur bone waste. Hybrid composite powders were prepared by adding varying amounts of MgO and MgO–graphene to the produced sheep hydroxyapatite powders and sintering them at 1200 °C for 5 min using induction sintering. The physical, mechanical, microstructural, in vitro bioactivity, cell culture, and antibacterial properties were studied. According to the results of the study, the density and compressive strength values of the samples containing 1 wt.% MgO and 1 wt.% MgO–0.1 graphene (KHM1 and KHM1GRF0.1), which had the best density and compressive strength values, were determined to be 2.771 g/cm^3^–28.42 MPa and 2.636 g/cm^3^–26.25 MPa, respectively. According to the in vitro bioactivity test in simulated body fluid, these composites exhibited bioactivity, with a dense hydroxy carbona apatite layer forming. Moreover, according to the cell culture and antibacterial test results, it was determined that sheep-derived hydroxyapatite, resulting from induction sintering with MgO and graphene, exhibited excellent biocompatibility, enhanced osteogenic potential, and moderate antimicrobial activity. In summary, these sheep hydroxyapatite hybrid composites exhibited higher mechanical strength and excellent integrated biological performance, confirming their substantial potential as advanced biomaterials for bone regeneration.

## 1. Introduction

In the field of biomaterials, autografts have long been used to repair bone defects. However, their use carries significant limitations due to donor site morbidity (such as pain, infection, and risk of fracture) [1,2]. Alternative allografts lose their osteoinductive properties and carry the potential for disease transmission or immune rejection due to their processing processes [3]. Tricalcium phosphates (TCP) are among the synthetic bone substitutes which are biocompatible, but tend to exhibit rapid degradation rates and low mechanical strength in load-bearing applications [4]. Hydroxyapatite is one of the most researched ceramic materials due to the high similarity of its chemical composition and crystal structure to the inorganic phase in natural bone tissue [5]. Hydroxyapatite has a wide range of applications, from orthopedic implants and dental fillings to tissue engineering scaffolds and coating materials, thanks to its high biocompatibility, osteoconductivity, bioinertness, and ability to directly bond with bone tissue [6]. However, the production of hydroxyapatite by conventional methods is not economically and environmentally sustainable due to the use of high-purity chemical precursors, the requirement of high temperatures and the generation of chemical waste. In recent years, with the rise in environmentally friendly production approaches, intensive studies have been conducted on the utilization of biological waste. Animal-derived bones, particularly bovine, porcine, and sheep bones, offer attractive alternatives for hydroxyapatite synthesis as natural resources rich in calcium and phosphate [7]. The use of such biological resources both provides an environmental solution for waste management and enables the cost-effective production of bioceramics. Sheep bone has significant potential for sustainable hydroxyapatite production due to its high calcium–phosphate ratio, low organic residue content, and easy accessibility in regions with intensive livestock farming. Hydroxyapatite production from sheep bone generally involves the removal of organic components, calcination, powder production, and sintering. Among these stages, sintering is the most critical step determining the microstructural, physical, and mechanical properties of hydroxyapatite [8]. Sintering temperature, time, and atmospheric conditions directly affect the density, porosity, crystalline structure, and phase stability of the material. Improper sintering conditions can lead to the transformation of hydroxyapatite into the tricalcium phosphate phase or a weakening of mechanical properties. Partial decomposition and a decrease in mechanical strength were observed in hydroxyapatite samples sintered above 1150 °C [9]. Therefore, determining the optimum sintering temperature is crucial for achieving high density and controlled porosity while maintaining phase stability. Although hydroxyapatite is widely used in bone tissue engineering due to its high biocompatibility and chemical similarity, it exhibits low densification capacity and limited mechanical strength in its pure form. Studies aimed at addressing these limitations have demonstrated the positive effects of additive oxides, particularly MgO, on sintering behavior. The addition of MgO displaces magnesium and calcium, creating crystal defects in the structure. This accelerates the diffusion mechanisms of hydroxyapatite during sintering, facilitating densification at lower temperatures [10,11]. In recent years, graphene has become one of the most widely studied reinforcement elements in the production of hydroxyapatite composites and coatings. Graphene consists of two-dimensionally arranged sheets with sp2-hybridized and strongly bonded carbon atoms in a honeycomb structure. It provides a unique combination of tensile strength (~100 GPa) and elastic modulus (~1 TPa). Studies have shown that graphene reinforcement offers superior mechanical properties compared to pure hydroxyapatite [12,13,14].

When hydroxyapatite is sintered using the conventional sintering method, the time required to reach high temperatures (>1000 °C), wait at the peak temperature, and cool to room temperature is very long (≈10 h). This sintering process also increases energy consumption. Furthermore, prolonged sintering at high temperatures can cause hydroxyapatite to transform into different phases (e.g., tricalcium phosphate), promote grain growth, and also damage the structure of reinforcing elements, such as graphene, used in composite production. Therefore, new and fast sintering methods that are low in energy and do not damage the crystal structure of the matrix and reinforcing elements are needed [15,16,17]. One such method is induction sintering, which offers several distinct advantages over conventional sintering techniques. Heating in induction sintering is provided by electromagnetic induction, resulting in very rapid and uniform heating of the composite in a few minutes [18,19]. This results in shorter sintering times, less grain growth, and improved microstructural homogeneity, which is particularly important for preserving the nanostructure and bioactivity of hydroxyapatite-based materials. Furthermore, localized and controllable heating minimizes the thermal degradation of hydroxyapatite and powders such as graphene, which often occurs during prolonged, high-temperature sintering [20].

In the literature, ceramics such as Al_2_O_3_ and YSZ are mostly sintered using induction. Oh et al. produced high-density materials by preventing grain growth through induction sintering of WC-Al_2_O_3_ [21]. Jeong et al. added graphene to 8YSZ and sintered it with induction; from the results, a nanoscale microstructure that prevents grain growth was observed. They also improved the fracture toughness of the 8YSZ-graphene composite [22]. In the literature, the number of studies on induction sintering of hydroxyapatite is limited. Khalil et al. reported the induction sintering of a magnesium-added hydroxyapatite composite. The nanostructure was preserved, and relative density, microhardness, and compressive strength values were determined [23]. Studies in the literature are mostly conducted using powders obtained commercially or synthesized in laboratories using harmful chemicals.

Due to these considerations, this study aims to produce sheep hydroxyapatite powder using a green process and create composites by processing MgO with MgO–graphene. Another purpose is to achieve a synergistic effect by combining the high mechanical properties of graphene with the sintering ability of MgO for hydroxyapatite hybrid composites. Therefore, this study is unique in that it is the first to specifically address the induction sintering of MgO–graphene hybrid hydroxyapatite composites using a green-process hydroxyapatite. In addition, the bioactivity, in vitro behavior and antibacterial properties of the environmentally friendly hybrid hydroxyapatite composites produced are among the unique aspects of the study.

## 2. Materials and Methods

### 2.1. Materials

In this study, sheep hydroxyapatite (KHA) was used as a matrix, while magnesium oxide (MgO, Nanografi, 99.9 purity, 325 micron) and graphene (Grafen Chemical Ind., 10 micron diameter, 5–8 nm thickness, Ankara, Türkiye) were used as reinforcement elements for composite production, the details of which are given below.

### 2.2. Production of Green Process-Based Hydroxyapatite Powders

**Figure 1:**
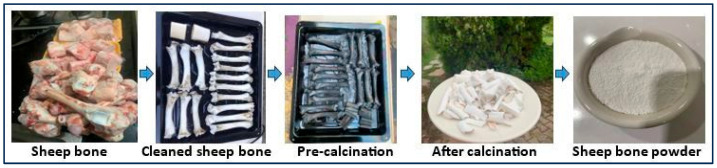
Hydroxyapatite production process with green process.

As shown in Figure 1, the sheep femur bone used as raw material for hydroxyapatite production was supplied by Ayvaz Et, a veterinary company in Türkiye. The mesoporous structure at the head and base of the sheep bones was excised, leaving the remaining bone structure in the middle. The cut bones were then stripped of soft tissue and cartilage. The prepared sheep femur blocks were first pre-cleaned by boiling them twice in hot water for 3 h to remove soft tissue such as marrow and fat. They were then boiled twice in a pressure cooker for 1 h each. The bones, completely stripped of their soft tissue, were boiled in a 4% NaOH solution by volume in a pressure cooker for 5–6 cycles of 30 min each. The boiling process was continued by replacing the NaOH each time. The bones were washed in running water and then soaked in clean water for 20 min to remove any remaining sodium hydroxide thoroughly. After the chemical cleaning, the bones were pre-calcined in an electric oven at 360 °C for approximately 3 h. The outer layer of fat on the bones was burned off, removing the organic matter. After the incineration process, the bone was subjected to a 4 h furnace at 800 °C to remove excess carbon. Thus, the carbon in the bone lost its stability, and the bones were ready to be converted into pure hydroxyapatite powder.

### 2.3. Fabrication and Characterization of Composites

Bone powders obtained from sheep femur were supplemented with MgO at 1%, 5%, and 10 wt.% and mixed in ethyl alcohol using a ball mill at 1000 rpm for 5 min for the production of Mgo-reinforced composites of sheep hydroxyapatite. The mixed powders were dried in an oven at 50 °C. After drying, the powders were crushed in an agate mortar and passed through a sub-100-micron sieve. To increase the formability of the powder, an aqueous solution containing 2.5% PVA was sprayed onto the powder surface, partially granulating it. Similarly, for hybrid composite production, graphene was added to the hydroxyapatite composite obtained from sheep femur at 0.1%, 0.5%, and 1 wt.% while maintaining a constant 1% MgO content. The resulting powders were pre-formed under 50 bar and then induction-sintered. The sintering temperatures were set at 1100, 1150, and 1200 °C, with different sintering times ranging from 0.5 to 2 min. The optimal density value was achieved for the KHO sample at 1200 °C and 1.5 min. Therefore, the induction sintering process was conducted at 1200 °C for 1.5 min using a graphite mold. The produced samples are hereafter coded as sheep hydroxyapatite KHO, MgO-reinforced composite KHMx (x = 1, 5, 10 wt.%), and hybrid composite KHM1GRFx (x = 0.1, 0.5, 1 wt.%).

The Archimedes method was used to determine the densities of the samples, and density and porosity were calculated with Equations (1) and (2). In Equation (1), P: density (g/cm^3^); k_1_: dry weight of the specimen (g); k_2_: weight of the specimen after liquid impregnation (g); k_3_: Suspended weight of the specimen in water (g); and ρw: density of water (g/cm^3^). In Equation (2), P_x_: porosity (%); d_m_: density of the specimen (g/cm^3^); and d_y_: density of hydroxyapatite

*P* = *ρ_w_ k*_1_/(*k*_3_ − *k*_2_) (1)

*P_x_* = (1 − (*d_m_/d_y_*)) ∗ 100 (2)

Compressive strength measurements of all prepared composites were taken on a universal testing machine with a head speed of 1 mm/min. The microstructures of the composites were examined using a scanning electron microscope (SEM, JEOL JSM-6060LV, JEOL Ltd., Tokyo, Japan). Crystal structure analysis was performed with an X-ray diffractometer (XRD, Rigaku Holdins Corp., Tokyo, Japan) at an angular range of 2θ = 20°–90°, at a speed of 2°/min.

### 2.4. Bioactivity Testing

Kokubo simulated body fluid was employed for bioactivity testing [24]. A 1000 mL solution was prepared using pure water and the components listed in Table 1. The temperature and pH were adjusted to 36.5 °C and 7.4. The solutions were divided into 250 mL containers, and the prepared samples were stored in these solutions for up to four weeks. The surface morphologies of the samples were examined using SEM and FTIR analysis.

### 2.5. Cell Culture Studies—Isolation and Culture of Mesenchymal Stem Cells

Powders were sterilized by autoclave and used in all experiments at a final concentration of 1 mg/mL. Adipose-derived mesenchymal stromal cells (aMSCs) were isolated from adipose tissue collected from adult male sheep immediately post-slaughter. The tissue was carefully dissected into small fragments to facilitate subsequent enzymatic digestion. The procedure employed Type IA collagenase (Sigma Aldrich, St. Louis, MO, USA) for 60 min at 37 °C to release the resident cells from the extracellular matrix. After digestion, the isolated cells were seeded into culture flasks containing Dulbecco’s Modified Eagle’s Medium (DMEM; Life Technologies, Paisley, UK) supplemented with 10% fetal bovine serum (FBS; Life Technologies, Paisley, UK). Cultures were maintained at 37 °C in incubators with 5% CO_2_, ensuring optimal conditions for cell growth.

### 2.6. MTT Assay Studies

An MTT assay was used to assess cell viability and mitochondrial metabolic activity in the presence of the different substrates. aMSCs at passage 2 were seeded in 24-well plates at a density of 40,000 cells/mL to ensure an even distribution and consistent proliferation. All substrates (KHO, KHM1, and KHM1GRF0.1) were weighed and then sterilized by autoclave at 121 °C for 20 min to ensure complete elimination of microbial contaminants. Thereafter, the materials were suspended in culture medium at a final concentration of 1 mg/mL. After an initial 24 h incubation period to allow cell adhesion, the culture medium was replaced with a medium containing the substrates under investigation.

Following an additional 24 h of incubation, the cells were treated with an MTT solution (0.5 mg/mL; Sigma Aldrich, St. Louis, MO, USA) and incubated for 3 h. During this time, the mitochondria of viable cells reduced the yellow tetrazolium salt (MTT) to insoluble, violet formazan. Subsequently, isopropanol (Sigma Aldrich, St. Louis, MO, USA) was added to solubilize the formazan, and the optical density of the resulting solution was measured at 600 nm using a BioPhotometer (Eppendorf, Hamburg, Germany). This measurement indirectly quantifies the number of viable cells and allows for the comparison of growth percentages with the control (cells cultured in the absence of a substrate).

### 2.7. Osteogenic Differentiation Studies

To evaluate the ability of the substrates to promote differentiation toward the osteogenic lineage, aMSCs at passage 2 were cultured under differentiative conditions. The cells were seeded in 6-well plates at a density of 50,000 cells/mL and allowed to adhere for 24 h in standard growth medium. Subsequently, the medium was replaced with an osteogenic medium composed of DMEM supplemented with 50 µg/mL ascorbic acid, 10 mM β-glycerophosphate, and 10^−7^ M dexamethasone. To this medium, the sterile substrate suspensions (KHO, KHM1, or KHM1GRF0.1) were added at a final concentration of 1 mg/mL for the entire duration of the experiment (21 days). During this period, the osteogenic medium was renewed every three days.

At the end of the experimental period, the cells were fixed with 4% paraformaldehyde (Sigma Aldrich, St. Louis, MO, USA) and stained with Alizarin Red S (Carlo Erba, Cornaredo, Italy), a dye that specifically binds to calcium deposits in the extracellular matrix, thereby providing a visual and quantifiable measure of mineralization. Photomicrographs of the various monolayers were captured using an inverted optical microscope (Nikon, Eclipse TE 2000-U, Nikon Instrument Inc., Amstelveen, The Netherlands). Quantification was achieved by treating the cultures with a solution of sodium dodecyl sulfate in HCl and measuring the optical density at 490 nm with a BioPhotometer (Eppendorf, Hamburg, Germany), comparing the data to that of the aMSC control.

### 2.8. Antimicrobial Activity Studies

The antimicrobial activity of the substrates was assessed by examining their inhibitory effects on five different microorganisms. Cultures of bacteria (*E. coli*, *S. aureus*, *P. aeruginosa*, *E. faecalis*) and the fungus *C. albicans* were incubated in Brain Heart Infusion (BHI; DIFCO, Sparks, USA) broth in the presence of the sterile substrates (KHO, KHM1, KHM1GRF0.1) at a final concentration of 1 mg/mL for 24 h. Incubation conditions were adjusted based on the requirements of the microorganisms: bacterial cultures were maintained at 37 °C, whereas the fungal culture was kept at 28 °C. Microbial growth was evaluated by measuring the optical density at 600 nm using a BioPhotometer (Eppendorf, Hamburg, Germany) and comparing the results with positive controls representing growth in the absence of any substrate.

### 2.9. Statistical Analysis

All experiments were performed in triplicate to ensure reproducibility and reliability of the data. The results are expressed as mean ± standard deviation (SD) and were statistically analyzed using the non-parametric Dunnett’s test, which allows for comparisons between each experimental group and the control group. Significance levels of *p* ≤ 0.05 *, *p* ≤ 0.01 **, and *p* ≤ 0.001 *** were considered, enabling the detection of even minor but significant differences between experimental conditions.

## 3. Results and Discussions

### 3.1. Composite Characterization

In many studies in the literature, hydroxyapatite has been produced via both chemical and natural routes and sintered using conventional methods. Sevostianov et al. synthesized hydroxyapatite powder using the precipitation method. After shaping, they sintered it at 1200 °C for 2 h. As a result, they obtained approximately 23% porosity and a compressive strength of 7.66 MPa [25]. Obada et al. produced hydroxyapatite from natural bone and achieved a compressive strength of 0.84 MPa after sintering at 1200 °C for 2 h [26]. Abifarin et al. obtained samples at different forming loads using hydroxyapatite powder of various sizes. These samples were sintered at 900 °C. The best compressive strength was found to be 3.25 MPa with a 6 kN forming load for a sample with a powder size of 100 microns [27]. The mechanical properties obtained from these studies are lower than those reported in our study, as shown in Table 2. Table 2 presents the MgO and MgO/graphene-added KHO composites at varying rates. The composite containing 1 wt.% MgO has the best density and compressive strength. Higher density and lower porosity directly improve the compressive strength of the composite. Low content of MgO plays a role in improving sintering within the KHO. Factors such as the formation of agglomerates in the structure with increasing MgO content, decreased particle neck formation due to the higher sintering temperature of MgO itself, and the presence of possible secondary phases have all contributed to reduced mechanical properties [28]. Hybrid composites were produced with varying graphene properties for the KHM1 sample, which exhibited the best mechanical properties. As shown in the table, the hybrid composite containing 0.1 wt.% graphene yielded the best results, with compressive strength decreasing as the graphene content increased. Graphene particles are more homogeneously distributed within the structure and located at grain boundaries at a lower graphene content. This led to increasing strength during deformation due to mechanisms such as load transfer, crack deflection, and crack bridging. Graphene agglomerates with increasing graphene content due to electrostatic attraction due to its nanostructure, leading to a loss of homogeneity in the structure. Furthermore, these graphene agglomerates cause the stacked graphene sheets to slip more easily under load, thereby decreasing their mechanical properties [29,30].

**Figure 2:**
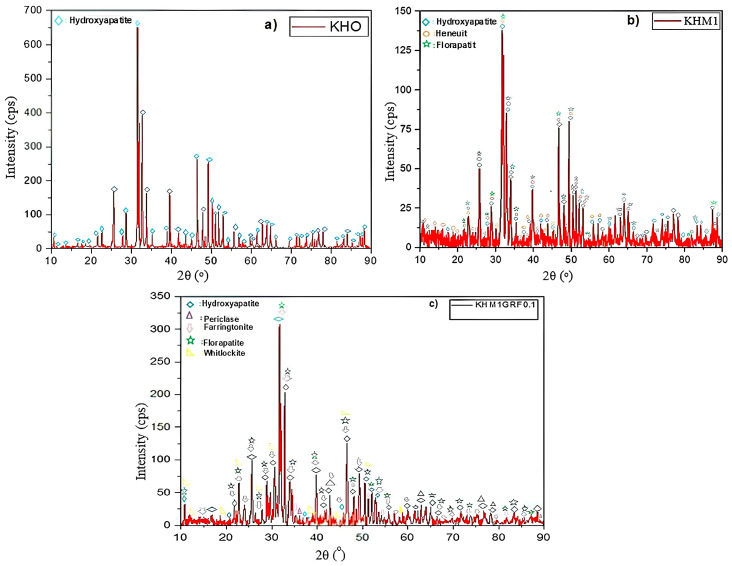
XRD graph of induction-sintered (**a**) KHO, (**b**) KHM1, and (**c**) KHM1GRF0.1 samples.

The X-ray diffraction pattern is shown in Figure 2a–c. It is observed that the hydroxyapatite phase was obtained without the addition of any additives (Figure 2a). Figure 2b gives the XRD of KHM1, and it is seen that hydroxyapatite, heneuite (CaMg_5_(CO_3_)(PO_4_)_3_(OH)), and fluorapatite ((Ca9.37Sr.63)(PO_4_)6F_2_) phases are formed. When studies on naturally produced hydroxyapatite are conducted, it is common to observe fluorapatite phases. The heneuite phase was formed as a result of the reaction between sheep hydroxyapatite and MgO. These phases positively contributed to the mechanical and physical properties of the composite. An XRD graph of the KHM1GRF0.1 hybrid composite is given in Figure 2c. The periclase (MgO), farringtonite (Mg_3_(PO_4_)_2_), hydroxyapatite (Ca_5_(PO_4_)_3_(OH)), fluorapatite (Ca5.061(P2.87O11.46)F0.89), and whitlockite Ca2.993H0.014(PO4)_2_ phases were observed. Substitution of Mg ions below 4 wt.% into HA prevents the formation of β-TCP, α-TCP, and CaO phases, enabling the formation of the whitlockite phase. The whitlockite phase, which constitutes 33.3% of the inorganic portion of human bone, was formed as a result of the reaction of composites prepared with 1% MgO and graphene added to sheep hydroxyapatite. Therefore, the coexistence of hydroxyapatite and whitlockite phases is a desirable property in biomedical applications [31,32,33,34].

**Figure 3:**
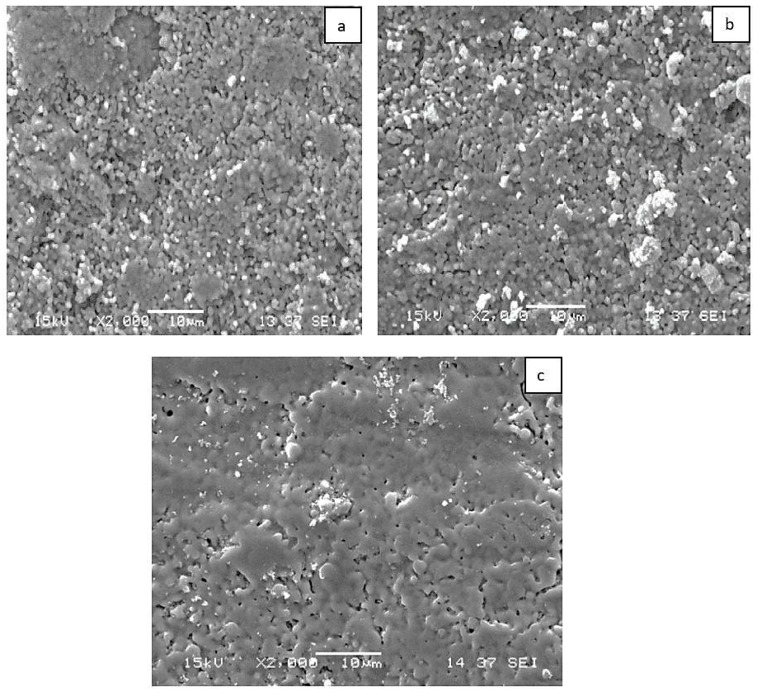
SEM images of induction-sintered (**a**) KHO, (**b**) KHM1, and (**c**) KHMGRF0.1 samples.

Figure 3a–c show the scanning electron microscope (SEM) images of the induction-sintered sheep hydroxyapatite and its composite form. The homogenous distributed porous structure of sheep hydroxyapatite is clearly visible. Additionally, when examining dense areas, it is observed that the grains are strongly bonded, particularly in the MgO-added samples. With the addition of MgO, some of the Ca^2+^ ions in the hydroxyapatite structure are replaced by Mg^2+^ ions. This displacement in the structure leads to crystal lattice stresses and structural defects. These crystal defects create diffusion pathways for atoms, accelerating mass transport during sintering. This causes the MgO-doped hydroxyapatite to densify at lower temperatures and inhibits grain growth [35,36].

### 3.2. Bioactivity Results

**Figure 4:**
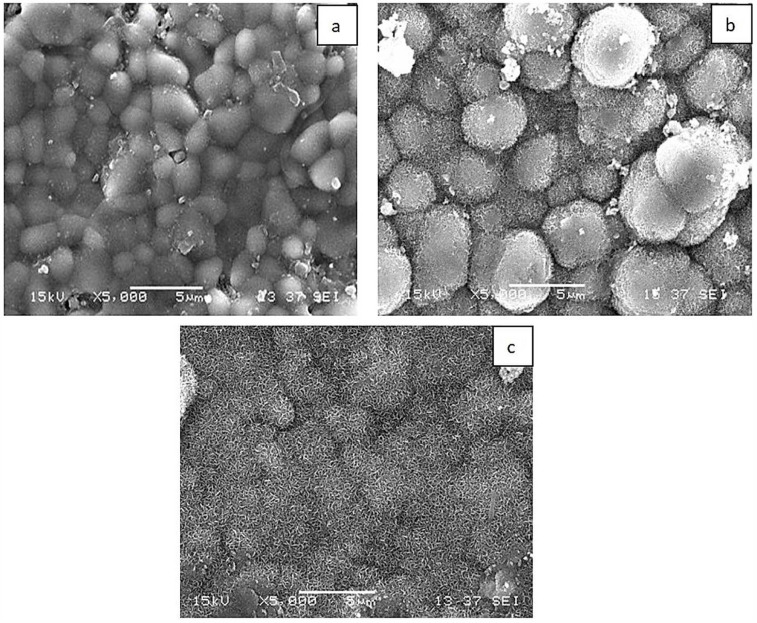
SEM analysis of (**a**) KHO, (**b**) KHM1, and (**c**) KHM1GRF0.1 composites.

Figure 4a–c show the in vitro bioactivity tests of KHO, KHM1, and KHM1GRF0.1 composites immersed in SBF for four weeks. When the SEM images of the graphene-added composites immersed in SBF were examined, it was observed that the apatite structure formation increased due to the presence of MgO and graphene, and denser hydroxy carbona apatite structures were formed, especially in the KHM1 and KHM1GRF0.1 composites.

### 3.3. MTT Assay

**Figure 5:**
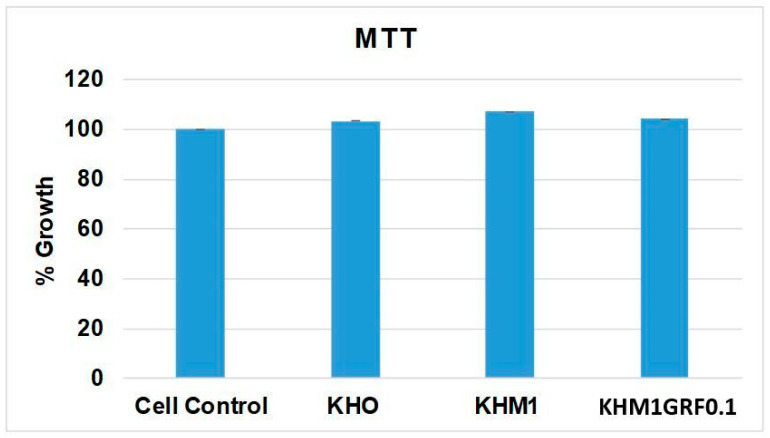
MTT assay showing the percentage of aMSC growth after 24 h in the absence (cell control) and in the presence of different substrates (KHO, KHM1, or KHM1GRF0.1). Data are expressed as mean ± SD from three independent experiments, with the control representing 100% growth.

The MTT assay served as the initial step to evaluate whether the substrates negatively affect aMSC viability. As shown in Figure 5, cells cultured in the absence of any substrate exhibited mitochondrial activity corresponding to 100% growth, establishing the benchmark for comparison. Exposure to KHO resulted in a cell growth percentage of approximately 103% relative to the control, indicating that KHO does not significantly alter cellular metabolism or proliferation. Cells with KHM1 exhibited a slightly higher proliferation rate, with growth reaching approximately 107%. This modest increase may be attributed to the beneficial effects of MgO, which, in addition to improving the structural properties of the material, may positively impact cellular metabolism. In the presence of the substrate containing graphene (KHM1GRF0.1), cells showed a growth value of about 104% compared to the control.

### 3.4. Osteogenic Differentiation

**Figure 6:**
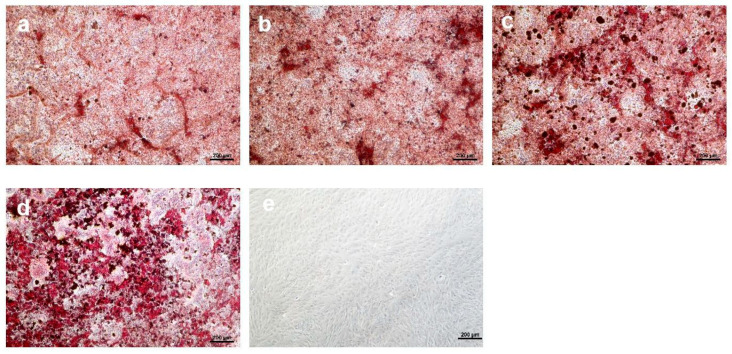
Alizarin Red S staining of aMSCs differentiated along the osteogenic lineage. (**a**) Positive control (aMSCs differentiated in the absence of substrates), (**b**) aMSCs differentiated in the presence of KHO, (**c**) aMSCs differentiated in the presence of KHM1, (**d**) aMSCs differentiated in the presence of KHM1GRF0.1, (**e**) aMSCs cultured in growth medium. Images were captured using an inverted optical microscope at 10× magnification.

**Figure 7:**
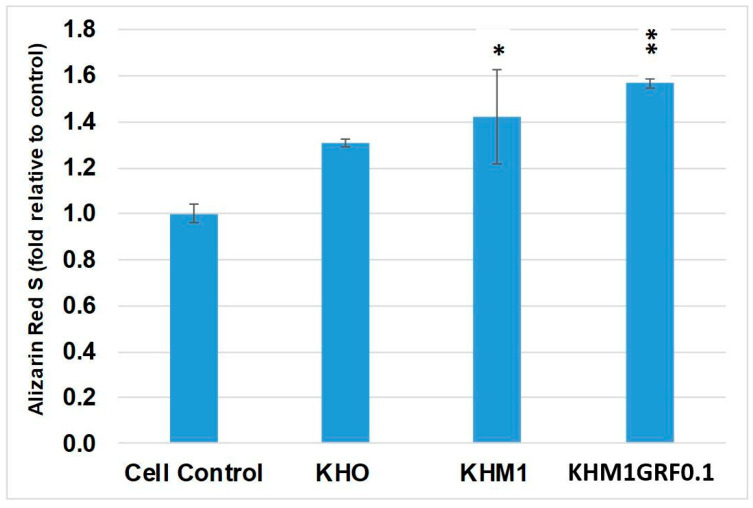
Photometric quantification of Alizarin Red S in aMSCs after osteogenic differentiation in the absence (positive control) and presence of substrates (KHO, KHM1, or KHM1GRF0.1). The values were obtained from three independent experiments and are expressed as mean percentage values ± SD, with the positive control set to 1. *p* values (Dunnett’s test): *p* ≤ 0.05 * and *p* ≤ 0.01 ** compared with the positive cell control.

The osteogenic process was evaluated by assessing calcium deposition within the extracellular matrix via Alizarin Red S staining. Figure 6 displays representative images of the various experimental conditions, and Figure 7 provides the photometric quantification of Alizarin Red S staining under these conditions.

aMSCs cultured under osteoinductive conditions without any substrate were assigned a reference value of 1-fold for calcium deposition. The addition of KHO to the medium increased calcium deposition, achieving a fold change of approximately 1.3 relative to the control. This suggests that KHO exerts a stimulatory effect on mineralization, likely by promoting matrix protein synthesis and activating specific osteoinductive pathways. Cells exposed to KHM1 exhibited an even greater increase, reaching approximately a 1.4-fold change (statistically significant, *p* ≤ 0.05) compared to the control, suggesting that the MgO component further facilitates the formation of calcium deposits. The fold change observed for cells treated with KHM1GRF0.1 was 1.5 (statistically significant, *p* ≤ 0.01).

### 3.5. Antimicrobial Activity

**Figure 8:**
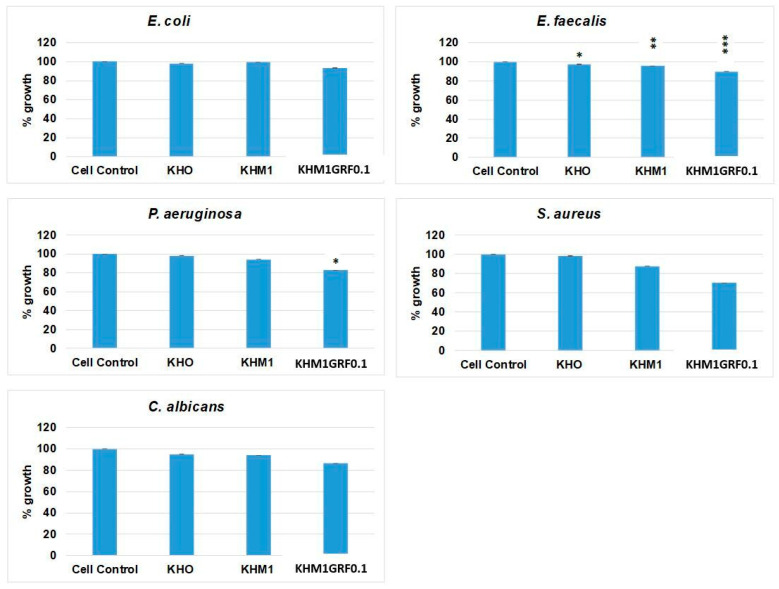
Antimicrobial activity of three substrates (KHO, KHM1, and KHM1GRF0.1) against *E. coli*, *E. faecalis*, *S. aureus*, *P. aeruginosa*, and *C. albicans*. The reported values were obtained from three independent experiments and are expressed as mean percentage values ± S.D. relative to the positive control value set at 100%. *p* values (Dunnett test): *p* ≤ 0.05 *, *p* ≤ 0.01 **, and *p* ≤ 0.001 *** compared to the control.

The efficacy of the substrates in inhibiting microbial growth was evaluated by incubating four bacterial strains and one fungal species in the presence of the materials (Figure 8). For E. coli, exposure to KHO, KHM1, and KHM1GRF0.1 resulted in low reduction in growth percentages (ranging from 2% to 7%) relative to the control. For E. faecalis, the reduction percentage values observed in the presence of KHO and KHM1 were 3% (statistically significant, *p* ≤ 0.05) and 5% (statistically significant, *p* ≤ 0.01), respectively, while treatment with KHM1GRF0.1 reduced the growth to 11% (statistically significant, *p* ≤ 0.001) relative to the control. The reduction in *P. aeruginosa* growth in the presence of KHM1GRF0.1 was pronounced (18%) and statistically significant (*p* ≤ 0.05) when compared to approximately 2% of KHO and 6% of KHM1. For S. aureus, treatment with KHO maintained nearly unchanged growth (approximately 98% with respect to 100% of control), while exposure to KHM1 reduced growth to 13%, and KHM1GRF0.1 produced a marked growth reduction to 30%. Similarly, for the fungus C. albicans, the substrates KHO and KHM1 exhibited modest inhibitory effects (resulting in 5% and 6% growth reduction, respectively), whereas KHM1GRF0.1 reduced growth to 14%.

The results from the MTT assay unequivocally demonstrate that all three substrates (KHO, KHM1, and KHM1GRF0.1) do not exhibit acute cytotoxicity at 24 h under the tested conditions. The ability of aMSCs to maintain proliferation comparable to that of the control group indicates that these materials do not induce cytotoxic effects or compromise cellular metabolism. This biocompatibility is an essential prerequisite for any regenerative medicine application, where cell survival and replication are crucial.

Furthermore, the calcium deposition assessed via Alizarin Red S staining indicates that the use of these substrates actively promotes osteogenesis. Notably, the most pronounced increase was observed with KHM1GRF0.1, suggesting that the combined presence of MgO and graphene plays a pivotal role in enhancing osteoinductive signaling. The inclusion of MgO appears to facilitate calcium deposit formation, while graphene contributes additional benefits without detracting from osteogenic potential.

The antimicrobial activity assays further underscore the potential clinical utility of these materials. Although KHO and KHM1 exhibited moderate inhibitory effects on microbial growth, the reduction in bacterial and fungal proliferation observed with KHM1GRF0.1 (particularly against *E. faecalis*, *P. aeruginosa*, and *S. aureus*) highlights the enhanced antimicrobial properties imparted by graphene.

This dual functionality—promoting bone regeneration while simultaneously reducing the risk of infection—is of considerable importance for implantable biomaterials in regenerative applications. Graphene and MgO additives are essential components that enhance osteogenic activity and antimicrobial efficiency in bone tissue engineering. Graphene promotes osteoblast differentiation by enhancing cell adhesion and protein adsorption, thanks to its high surface area and electrical conductivity [37]. On the other hand, MgO stimulates cell proliferation and mineralization through the controlled release of magnesium ions and exhibits antimicrobial effects [38]. Compared to the recently developed NIR-responsive 4D-printed polymeric systems, graphene–MgO composites provide a more sustained and direct biological effect. In 4D systems, osteogenic and antibacterial activities are achieved through NIR-triggered transient photothermal heating and shape adaptability [39], whereas in graphene–MgO systems, these effects arise intrinsically from the material itself. Therefore, graphene–MgO-based systems offer significant advantages over 4D polymeric scaffolds due to their long-term biostimulation, persistent antimicrobial activity, and relatively high mechanical strength.

In conclusion, the combined data support the hypothesis that modifying sheep-derived hydroxyapatite (HA) through the incorporation of MgO and graphene, along with high-temperature treatment (1200 °C), results in advanced biomaterials with excellent biocompatibility, enhanced osteogenic potential, and moderate antimicrobial activity. Such multifunctional substrates hold great promise for future applications in bone tissue engineering and regenerative medicine, offering a novel approach to both promoting tissue regeneration and mitigating infection risks. Further studies are necessary to elucidate the underlying molecular mechanisms and to explore the broader applicability of these materials in clinical settings.

## 4. Conclusions

In this study, eco-friendly sheep hydroxyapatite powder was fabricated at a lower cost from sheep femur bone waste. Varying amounts of MgO and MgO–graphene were added to the fabrication of hydroxyapatite powder and sintered with the induction method to obtain hybrid composites. From the results, the samples containing 1 wt.% MgO and 1 wt.% MgO–0.1 graphene (KHM1 and KHM1GRF0.1) had the best density and compressive strength, which were found to be 2.771 g/cm^3^–28.42 MPa and 2.636 g/cm^3^–26.25 MPa, respectively. These composites showed bioactivity due to the hydroxy carbon apatite film from the simulated body fluid test. The results from the MTT experiment showed that all three materials (KHO, KHM1, and KHM1GRF0.1) were biocompatible. Furthermore, calcium accumulation, assessed by Alizarin Red S staining, indicated that the use of these materials actively promoted osteogenesis. Specifically, the most significant increase was observed in the hybrid KHM1GRF0.1 combined with graphene and MgO. KHO and KHM1 exhibited moderate effects on microbial growth. The graphene-containing hybrid KHM1GRF0.1 composites were particularly effective against *E. faecalis*, *P. aeruginosa*, and *S. aureus.* These hybrid composites are a great candidate for future applications in bone tissue engineering and regenerative medicine to promote tissue regeneration and reduce infection risks. Although this study demonstrates some significant mechanical and in vitro biological potential of the hybrid composites, in vivo validation has not yet been performed. Also, the dynamic fatigue strength of the composites and their long-term degradation kinetics in the body can be studied in the future.

## Figures and Tables

**Table 1:** Simulated body fluid components.

Component	Amount
NaCI	11.994 g
NaHCO_3_	0.525 g
KCI	0.336 g
K_2_HPO_4_·3H_2_O	0.342 g
MgCl_2_6H_2_0	0.458 g
1 M-HCI	60 mL
CaCI_2_	0.417 g
Na_2_SO_4_	0.107 g
NH_2_C(CH_2_OH)_3_	9.086 g

**Table 2:** Density, porosity, and compressive strength of KHO and its composite form.

Sample Code	Density (g/cm^3^)	Porosity (%)	Compressive Strength (MPa)
KHO	2.36 ± 0.26	26.18 ± 0.26	7.50 ± 2.14
KHM1	2.77 ± 0.18	12.30 ± 0.18	28.42 ± 4.22
KHM5	2.46 ± 0.22	22.12 ± 0.22	18.89 ± 2.83
KHM10	2.54 ± 0.20	19.31 ± 0.20	12.05 ± 3.46
KHM1GRF0.1	2.63 ± 0.16	16.48 ± 0.16	26.25 ± 4.21
KHM1GRF0.5	2.55 ± 0.24	19.30 ± 0.24	19.03 ± 3.71
KHM1GRF1	2.46 ± 0.19	22.15 ± 0.19	18.68 ± 3.25

## Data Availability

The original contributions presented in this study are included in the article. Further inquiries can be directed to the corresponding author.
